# Impact of Composition and Autoclave Sterilization on the Mechanical and Biological Properties of ECM-Mimicking Cryogels

**DOI:** 10.3390/polym16131939

**Published:** 2024-07-07

**Authors:** Laura Di Muzio, Susi Zara, Amelia Cataldi, Claudia Sergi, Vito Cosimo Carriero, Barbara Bigi, Simone Carradori, Jacopo Tirillò, Stefania Petralito, Maria Antonietta Casadei, Patrizia Paolicelli

**Affiliations:** 1 Department of Drug Chemistry and Technologies, Sapienza University of Rome, 00185 Rome, Italy; laura.dimuzio@uniroma1.it (L.D.M.); vitocosimo.carriero@uniroma1.it (V.C.C.); barbara.bigi@uniroma1.it (B.B.); stefania.petralito@uniroma1.it (S.P.); mariaantonietta.casadei@uniroma1.it (M.A.C.); 2 Department of Pharmacy, University “G. d’Annunzio” Chieti-Pescara, 66100 Chieti, Italy; susi.zara@unich.it (S.Z.); amelia.cataldi@unich.it (A.C.); simone.carradori@unich.it (S.C.); 3 Department of Chemical Engineering Materials Environment, Sapienza University of Rome, 00184 Rome, Italy; claudia.sergi@uniroma1.it (C.S.); jacopo.tirillo@uniroma1.it (J.T.)

**Keywords:** cryogels, chondroitin sulfate, gelatin, macroporous networks, scaffolds, tissue engineering, cell adhesion

## Abstract

Cryogels represent a valid strategy as scaffolds for tissue engineering. In order to adequately support adhesion and proliferation of anchorage-dependent cells, different polymers need to be combined within the same scaffold trying to mimic the complex features of a natural extracellular matrix (ECM). For this reason, in this work, gelatin (Gel) and chondroitin sulfate (CS), both functionalized with methacrylic groups to produce CSMA and GelMA derivatives, were selected to prepare cryogel networks. Both homopolymer and heteropolymer cryogels were produced, via radical crosslinking reactions carried out at −12 °C for 2 h. All the scaffolds were characterized for their mechanical, swelling and morphological properties, before and after autoclave sterilization. Moreover, they were evaluated for their biocompatibility and ability to support the adhesion of human gingival fibroblasts and tenocytes. GelMA-based homopolymer networks better withstood the autoclave sterilization process, compared to CSMA cryogels. Indeed, GelMA cryogels showed a decrease in stiffness of approximately 30%, whereas CSMA cryogels of approximately 80%. When GelMA and CSMA were blended in the same network, an intermediate outcome was observed. However, the hybrid scaffolds showed a general worsening of the biological performance. Indeed, despite their ability to withstand autoclave sterilization with limited modification of the mechanical and morphological properties, the hybrid cryogels exhibited poor cell adhesion and high LDH leakage. Therefore, not only do network components need to be properly selected, but also their combination and ability to withstand effective sterilization process should be carefully evaluated for the development of efficient scaffolds designed for tissue engineering purposes.

## 1. Introduction

In native tissues, cells are surrounded by a complex and heterogenous cell niche. Each cell niche is composed of a specific geometrical, topographical and molecular architecture, giving rise to an individual footprint. Crucial cell niche factors comprise molecular components, topography, geometry and stiffness of the extracellular matrix (ECM). Cells sense these individual properties via transmembrane receptors like ion channels, cell–cell contacts or cell–matrix contacts, and subsequently activate intracellular cascades that tune specific biological responses, such as the cytoskeletal structure and nuclear import of transcription factors. This relationship between cells and their structural environment ultimately drives cellular fate decisions like cell migration, differentiation or proliferation [1]. Thus, to control the fate decisions of cells and tissue formation, scaffolds used for tissue engineering purposes should be able to mimic the complex physicochemical properties of the cellular microenvironment. For these reasons, their design must take into consideration proper selection of the constitutive polymers to attain adequate cell adhesion, migration, differentiation and proliferation [2]. Indeed, ECM-mimicking structures potentially provide the cells with the biological cues found in native tissues [3,4,5]. In this work, the effect of the scaffold composition on the mechanical and biological responses was investigated in vitro, starting from the recently reported results on cryogels made of methacryloyl derivatives of dextran (DexMA) and gelatin (GelMA) [6]. The cryogel composition was modified and optimized to make the scaffold closely similar to native ECM. Indeed, an ideal scaffold should mimic native tissues with respect to physical, biological and mechanical properties [7]. Generally, a single biomaterial hardly allows all these features to be faithfully reproduced and to recapitulate tissue microenvironment [8,9]. Therefore, a suitable polymer combination should be used for scaffold production along with an efficient fabrication technique, so that a particular cell type can efficiently proliferate and differentiate within the scaffold [10,11]. In particular, GelMA is frequently blended with glycosaminoglycans (GAGs), which, as ECM components, are able to drive cell fate and tissue regeneration. However, each GAG has been investigated to a different extent [12]. While much work has been done with hyaluronic acid, blending of GelMA with chondroitin sulfate (CS) in cryogel-type scaffolds has been less investigated, even though CS can represent an interesting option for the regeneration of specific tissues, such as cartilage [13].

For these reasons, in this work, a methacrylate derivative of chondroitin sulfate (CSMA) was used in combination with GelMA. As a collagen derivative, Gel comprises the arginine–glycine–aspartic acid (RGD) sequences and degradable motifs of matrix metalloproteinase (MMP). The RGD sequence fosters the growth of cells, while the MMP motifs regulate the enzymatic tissue remodeling. Such sequences are highly preserved in GelMA-based scaffolds, thus making them appropriate for tissue engineering and wound healing applications [14]. Chondroitin sulfate (CS), instead, is a GAG mainly found in connective tissue, bones and cartilage, and forms an important component of cartilage, where it plays a key role in chondrogenesis by providing signals for chondrocyte proliferation, migration and differentiation [15,16,17]. Moreover, CS provides resistance to compression, which in turn leads to increased scaffold strength, which is another important feature for efficient regeneration of load-bearing tissues [18,19]. Indeed, mechanical strength ensures that the scaffold can withstand mechanical load, when it is implanted in the joint as tissue replacement. It also provides a strong ground for chondrocytes so that they can retain their phenotype [20,21]. 

The two polymers, namely GelMA and CSMA, were used to prepare homopolymer and heteropolymer macroporous scaffolds of the cryogel type. The effect of the composition and architecture on the morphology of the scaffolds was investigated, together with the effect on the swelling and mechanical properties. These features were investigated before and after sterilization, in order to evaluate the impact of the process on the suitability of the cryogels as scaffolds for tissue engineering purposes. Although sterility is a fundamental requirement for any biomaterial intended for biomedical application, the effects of sterilization procedures on the intrinsic properties of polymer networks, such as cryogels, are frequently understudied [22]. Of all the methods available for sterilization, high-pressure steam sterilization, such as autoclaving, is the most effective in obtaining an established sterility assurance level. While autoclave sterilization minimizes pathogen contamination, it can dramatically impact both structural and biological properties of biomaterials [23,24]. For this reason, in this study, the impact of autoclave sterilization on structural and physical properties of a series of ECM-mimicking cryogels was investigated, in order to engineer robust and mechanically stable scaffolds capable of sustaining autoclaving, while retaining their unique physicochemical features. Then, the biocompatibility of the different scaffolds, as well as their ability to efficiently support cell adhesion, was investigated using primary human gingival fibroblasts (HGFs) and tenocytes for the scope. Indeed, tenocytes and fibroblasts represent the most abundant cell populations in tendons and connective tissue, respectively, and possess a key role in determining the architecture of connective tissues and joints. Additionally, as widely demonstrated [25], the healing process occurring in an injured site of both connective tissue and joints is characterized by a fibroblastic phase involving a high rate of migration of fibroblasts before and after proliferation.

## 2. Materials and Methods

### 2.1. Materials

All used reagents were of analytical grade. Type A gelatin (Gel) from porcine skin (~300 bloom), chondroitin sulfate A sodium salt, anhydrous dimethyl sulfoxide (DMSO), deuterated dimethyl sulfoxide (DMSO-d_6_), deuterated water (D_2_O), methacrylic anhydride (MAA), nicotinamide (Nic), ammonium peroxydisulfate (APS), N,N,N′,N′-tetramethylethylenediamine (TEMED), glycidyl methacrylate (GMA), 4-dimethyl aminopyridine (DMAP), tetrabutylammonium bromide (TBAB), sodium chloride, dialysis membranes (cut-off 12–14 kDa) and L-glutamine were purchased from Merck, (Milan, Italy). Spongostan^TM^ Dental was purchased from Ethicon (Somerville, NJ, USA). Absolute ethanol (EtOH), 37% *w*/*w* hydrochloric acid (HCl), monobasic potassium phosphate (KH_2_PO_4_) and sodium hydroxide in pellets (NaOH) were purchased from Carlo Erba (Milan, Italy). Vybrant^®^ CDFA SE cell tracer kit, alamarBlue™ cell viability reagent and Primocin^TM^ were purchased from Invitrogen (ThermoFisher Scientific, Waltham, MA, USA) Fetal Bovine Serum (FBS) from Gibco (Buffalo, NY, USA), Dulbecco’s Phosphate Buffered Saline (PBS, 1X) from Aurogene (Rome, Italy), DMEM, alpha MEM culture medium and penicillin–streptomycin solution (10,000 U/mL) from Euroclone (Milan, Italy).

### 2.2. Synthesis and Characterization of Methacryloyl Gelatin (GelMA)

GelMA was synthesized following a single-phase procedure reported in the literature [26]. Briefly, Gel was solubilized in anhydrous DMSO at the concentration of 5% *w*/*v* (1 g in 20 mL) for 3 h at 50 °C, under magnetic stirring. After complete solubilization, 0.3 mL of MAA was added and the solution was kept under magnetic stirring at 50 °C for 3 h. Then, it was dialyzed exhaustively against deionized water at 37 °C for 3 days and freeze-dried. The obtained polymer was characterized by ^1^H NMR using a Bruker AC-400 spectrometer (MA, USA). To this end, 10 mg of GelMA were dissolved in 0.5 mL of D_2_O, and then 0.2 mL of a solution of the internal standard nicotinamide (1 mg/mL in D_2_O) were added. The derivatization degree (DD) of GelMA was 0.53 ± 0.02 mmol/g.

### 2.3. Synthesis and Characterization of Chondroitin Sulfate Methacrylate (CSMA)

The synthesis of CSMA was carried out following a two-step procedure reported in the literature [27]. The first step was based on the preparation of the tetrabutylammonium salt of CS (CS^−^TBA^+^), in order to increase the solubility of the polymer in DMSO. To this end, Dowex^®^ resin (10 mL) was flushed with 20 mL of a TBAB aqueous solution (0.4 g/mL) and then washed with distilled water (100 mL) to remove the excess of the salt. Next, 10 mL of an aqueous solution of native CS (0.1 g/mL) was eluted through the resin and collected as CS^−^TBA^+^. Then, the polymer solution was frozen and freeze-dried. The freeze-dried product (10 mg) was dissolved in DMSO-d_6_ (0.7 mL) and analyzed through ^1^H NMR, using a Bruker AC-400 spectrometer, to determine the exchange degree (ED) of CS^−^TBA^+^. The ED was expressed as the content of TBA^+^ ions per disaccharide repeating unit of CS and was calculated according to the literature [28] and following Equation (1).

(1) $$ED=\frac{\;Average\;(I_{1.57},I_{1.32})\;}{8}:\frac{I_{1.77}}{3}$$

*I*_1.57_ and *I*_1.32_ indicate the integrals of the signals at 1.32 and 1.57 ppm, corresponding to the eight chemically equivalent methylene protons of the four aliphatic chains of TBA^+^ [N^+^(CH_2_C*H_2_*C*H_2_*CH_3_)_4_]. *I*_1.77_ represents the integral of the methyl peak of the acetamide group of CS at 1.77 ppm. Following the reported procedure, an ED of 1.0 ± 0.2 was obtained. 

To synthesize CSMA, the obtained CS^−^TBA^+^ (444 mg, 0.6 mmoles of disaccharide repeating units) was dissolved in anhydrous DMSO (8 mL) at 50 °C and, after complete dissolution, 4-DMAP (147 mg, 1.2 mmoles) and GMA (213 mg, 1.5 mmoles) were added to the polymeric solution, and the system was allowed to react at 50 ± 1 °C under magnetic stirring and N_2_ atmosphere for 24 h. After the reaction time, the mixture was diluted with 45 mL of distilled water and the pH was adjusted to 5.5 with HCl 2 M. The product was extensively dialyzed, first against 1 M NaCl for 2 days, then against distilled water for additional 2 days. Finally, the purified polymer was frozen and freeze-dried. The DD of the obtained CSMA was determined by ^1^H NMR. To this end, 10 mg of CSMA was dissolved in 0.5 mL of D_2_O, and then 0.2 mL of the internal standard nicotinamide (1 mg/mL in D_2_O) was added. Following the reported procedure, CSMA with a DD% of 25 ± 1 was obtained. 

### 2.4. Cryogel Preparation

Homopolymer and heteropolymer cryogels were synthesized by free radical crosslinking reaction, using APS and TEMED, as redox initiation system. To prepare GelMA cryogels, 120 mg of GelMA was solubilized in 1.750 mL of distilled water at 50 ± 1 °C under mild magnetic stirring for 15 min. Then, APS (160 μL, 5% *w*/*v*) and TEMED (90 μL, 5% *w*/*v*) were added and quickly mixed. After mixing, the polymer solution was placed and maintained in a cryostatic bath M408-BVC (MPM Instruments, Monza and Brianza, Italy) at −12.0 ± 1.0 °C for 2 h. After this time, the samples were freeze-dried to remove the ice crystals and obtain the corresponding GelMA scaffolds. To prepare CSMA cryogels, 120 mg of CSMA was solubilized in 1.795 mL of distilled water at room temperature under mild magnetic stirring for 15 min. Then, APS (131 μL, 5% *w*/*v*) and TEMED (74 μL, 5% *w*/*v*) were added and quickly mixed. After mixing, the polymer solution was placed and maintained in the cryostatic bath at −12.0 ± 1.0 °C for 2 h. After this time, the samples were freeze-dried to remove the ice crystals and obtain the corresponding CSMA scaffolds. Following this procedure, heteropolymer cryogels were also produced, dissolving 60 mg of GelMA and 60 mg of CSMA in 1.790 mL of distilled water under mild magnetic at 50 ± 1 °C stirring for 15 min. Then, APS (134 μL, 5% *w*/*v*) and TEMED (76 μL, 5% *w*/*v*) were added and quickly mixed. After mixing, the polymer solution was placed and maintained in the cryostatic bath at −12.0 ± 1.0 °C for 2 h. After this time, the samples were freeze-dried to remove the ice crystals and obtain the corresponding GelMA/CSMA scaffolds. In all the cases, the total volume of the polymer solutions was kept constant at 2 mL. All the cryogel samples were prepared in cylindrical glass molds (diameter 20 mm, height 40 mm).

### 2.5. Turbidity Measurements

Turbidity of aqueous solutions of GelMA (3 and 6% *w*/*v*), CSMA (3 and 6% *w*/*v*) and GelMA/CSMA (1:1 weight ratio and 6% *w*/*v* total polymer concentration) was evaluated by measuring transmittance values at 600 nm with a lambda 40 UV/Vis spectrophotometer (Perkin Elmer, Waltham, MA, USA).

### 2.6. Physical Characterization of Cryogels

#### 2.6.1. Compression Tests

The compressive properties of cryogels were evaluated, before and after the sterilization process, through a universal testing machine Zwick/Roell Z010 (Ulm, Germany) equipped with a 10 kN load cell employing a test speed of 1 mm/min. More specifically, cryogels were tested as prepared (neat samples), after swelling, washing and freeze-drying (refined samples) and after swelling, sterilization and freeze-drying (sterilized samples). All the samples were analyzed in dry conditions and after hydration.

#### 2.6.2. Field-Emission Scanning Electron Microscopy

The morphology of cryogels was evaluated through a field-emission scanning electron microscope (FE-SEM) MIRA 3 by Tescan (Brno, Czech Republic). Due to their low electrical conductivity, all specimens were sputter-coated with a thin layer of gold to prevent charging. The coating process was carried out in vacuum conditions (0.4 mbar) for 2 min by a sputter coater Edwards S150B applying a voltage of 1 kV and an electrical current of 40 mA to the gold electrode. Cryogels were analyzed as prepared (neat samples), after swelling, washing and freeze-drying (refined samples) and after swelling, sterilization and freeze-drying (sterilized samples).

#### 2.6.3. Porosity Evaluation

The porosity of cryogels in the dry state was characterized by image analysis of SEM micrographs. To this end, SEM micrographs were analyzed with the image processing software Image J ver. 1.53k to evaluate the pore size distribution. In particular, the micrographs were subjected to a thresholding process, that is, an image segmentation that converts from color or grayscale in a binary image. This allows us to highlight and select specified areas of interest of an image, thus making it possible to select singularly the pores which, once pinpointed, can be measured automatically by the program. A total number of 200 pores was measured to obtain a statistically significant distribution. 

The porosity in the swollen state was analyzed by investigating the change in weight based on the amount of fluid absorbed within the cryogel polymeric network [29]. Briefly, the samples were soaked in PBS (pH 7.4) and allowed to swell until they reached an equilibrium of swelling. The soaked formulations were weighed. Subsequently, the excess of PBS was removed by mechanical compression, and the squeezed gels were weighed again. The porosity was calculated with Equation (2).

(2) $$Porosity=\frac{W_{swollen\;gel}-W_{squeezed\;gel}\;}{W_{swollen\;gel}}\times 100$$

where W_swollen gel_ represents the weight of the swollen sample, while W_squeezed gel_ represents the weight of the gel after removal of the excess of PBS. The results are reported as the mean values ± the standard deviation.

#### 2.6.4. Swelling and Degradation Studies

The swelling degree of cryogels was determined in PBS. To this end, freeze-dried cryogels were weighed and then placed in an excess volume of PBS at 37.0 ± 0.5 °C. At established time points, the samples were taken and the excess of PBS gently wiped off before weighing. The swelling degree (Q) was calculated with Equation (3).

(3) $$Q=\frac{W_{s}}{W_{d}}$$

where W_s_ and W_d_ were the weights of the swollen and dry sample, respectively. The process of swelling was monitored up to the complete degradation of the cryogels. Each experiment was performed in triplicate, and the results were reported as the mean values ± the standard deviation.

#### 2.6.5. Effects of Sterilization on Cryogel Properties

Some cryogel samples were steam-sterilized. To this end, freeze-dried cryogels were hydrated in distilled water and then autoclaved at 121 °C for 10 min. At the end of the sterilization process, cryogels were frozen and freeze-dried. The obtained samples were submitted to compression tests and observed through SEM, to evaluate the effect of the sterilization process on the mechanical and morphological properties of the scaffolds. Moreover, sterilized cryogel samples were used for cell culture. 

### 2.7. Cell Cultures 

Cryopreserved adult tenocytes were purchased from Zenbio (Durham, NC, USA) and cultured in alpha MEM medium supplemented with 1% glutamine, 1% penicillin/streptomycin and 10% of fetal bovine serum (FBS). 

Primary HGFs were extracted from gingiva biopsies as previously reported [30]. The project has been approved by the Local Ethics Committee of the University of Chieti (approval number 1173, 31 March 2016), in agreement with the Declaration of Helsinki. All the donors signed informed consent before dental surgical extractive procedures. HGFs were cultured in DMEM medium with 1% glutamine, 1% penicillin/streptomycin and 10% of FBS.

GelMA, CSMA, GelMA/CSMA and Spongostan scaffolds were introduced into a 96-well plate treated for cell culture. On each scaffold, 6700 tenocytes or HGFs were seeded in drops of 60 µL and left to adhere for 6 h. After that, to quantify only signals derived from cells adhered on the scaffold surfaces, each sample was moved into a new sterile 96-well plate not treated for cell culture and there cultured up to 72 h at 37 °C within an incubator in the presence of 5% CO_2_. At the established time points (48 and 72 h), samples were processed for MTS and SEM analyses, and supernatants collected and frozen at −80 °C, for further analyses.

#### 2.7.1. Metabolic Activity Assay (MTS Assay)

The cell metabolic activity was estimated after 48 and 72 h of culture by MTS assay (Merck, Darmstadt, Germany). The method is based on the reduction of MTS tetrazolium compound by viable cells to generate a colored formazan derivate soluble in culture media. The conversion is performed by NAD(P)H-dependent dehydrogenase enzymes in metabolically active cells.

At established experimental times, the medium was removed and a fresh one with 10% MTS was added in each well; the plate was incubated for 4 h at 37 °C. The absorbance was spectrophotometrically read at 490 nm wavelength by Multiscan GO microplate reader (Thermo Fisher Scientific, Waltham, MA, USA). Three independent experiments were performed. 

#### 2.7.2. Lactate Dehydrogenase (LDH) Cytotoxicity Assay

To quantify the cytotoxic effect of the aforementioned scaffolds on tenocytes and HGFs, the LDH release in the culture medium, by means of CytoTox 96 Non-Radioactive Assay (Promega Corporation, Fitchburg, WI, USA), was detected. Fifty μL of supernatants were pipetted into a flat-bottom 96-well plate (Falcon, Corning Incorporated, New York, NY, USA) and 50 μL of the LDH reaction mixture was added. The plate was incubated for 30 min at room temperature in the dark, after which 50 μL of stop solution was added. The absorbance was read at 490 and 690 nm wavelength by means of Multiscan GO microplate reader. Released LDH was expressed, as elsewhere reported [31], as % LDH leakage and determined by applying Equation (4).

(4) $$\%\;LDH\;leakage=\left(\frac{A}{B}\right)\times 100$$

A: OD measured in treated sample
B: OD measured in lysed cells sample (maximum LDH activity)

#### 2.7.3. Collagen Type I ELISA

Human Collagen Type 1 ELISA detection kit (Cosmo Bio Co., Ltd., Tokyo, Japan) was applied to reveal the concentration of collagen type I (Col I), released by tenocytes, cultured on the aforementioned scaffolds in the culture medium, following the manufacturer’s instructions. The absorbance was spectrophotometrically read at 450 nm wavelength through Multiskan GO reader. The concentration of Col I, expressed as μg/mL, was obtained by plotting optical density (O.D.) values on a standard curve and then normalizing them with the MTS O.D. 

### 2.8. Scanning Electron Microscopy Analysis of Cell Layer on Cryogels

SEM analysis was performed to evaluate the adhesion and the confluence state of tenocytes on the above-reported scaffolds. Cells were fixed with 1.25% glutaraldehyde in 0.1 M cacodylate buffer at pH 7.2 for 30 min at 4 °C. Then, samples were dehydrated through the ascending alcohol series and dried with hexamethyldisilazane. Images were acquired by scanning electron microscope (SEM) Phenom XL at 15 kV accelerating conditions, in high vacuum ambient (1 Pa), beam spot size 5 μm (Thermo Fisher Scientific, Eindhoven, The Netherlands).

### 2.9. Statistical Analysis

Prism 5.0 software (GraphPad, San Diego, CA, USA) was used to carry out statistical analysis by applying one-way ANOVA, followed by post hoc test. Statistically significant values were established for *p* < 0.05.

## 3. Results and Discussion

**Figure 1:**
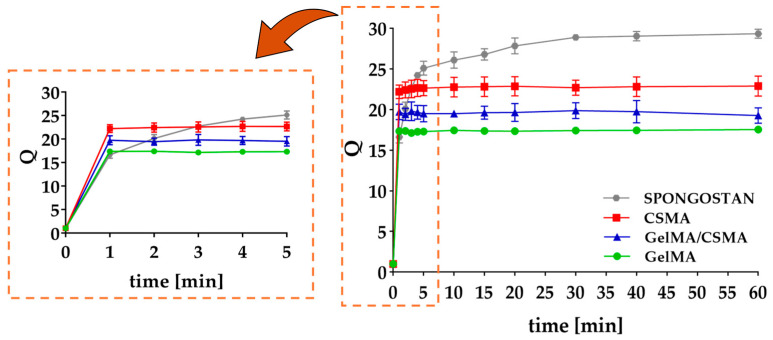
Swelling behavior of homopolymer (GelMA and CSMA) and heteropolymer (GelMA/CSMA) cryogels, in comparison with commercial Spongostan, used as a control. Swelling studies were performed in PBS (pH = 7.4) at 37.0 ± 0.5 °C.

The three key elements of tissue engineering include stem cells, inductive morphogenetic signals in an environment conductive to regeneration of a vital and functional tissue and/or organ and, finally, a scaffold. The latter element of the tissue engineering triad may be able to provide structural support for cell attachment and proliferation, mimicking as much as possible the ECM, which consists of collagen and proteoglycans [32]. This work was focused on the last element of the tissue engineering triad, selecting Gel and CS as base biomaterials for the scaffold fabrication. More specifically, these biopolymers were functionalized with methacryloyl groups, giving GelMA and CSMA respectively, to allow the formation of chemically crosslinked networks. To date, GelMA and CSMA blending in cryogel-type scaffolds has been scarcely investigated, even though CS can represent an interesting option for the regeneration of specific tissues, such as cartilage. Indeed, Han et al. reported superiority of cryogels made of GelMA and CSMA in cartilage tissue engineering, compared to scaffolds obtained blending GelMA with methacrylated hyaluronic acid [33]. However, the procedures used by Han et al. to synthesize GelMA and CSMA were not optimized and the derivatives obtained were poorly characterized, thus introducing high variability in scaffold architecture and consequently in the biological response [34,35]. Therefore, in this work, GelMA and CSMA were synthesized according to well-established protocols able to provide derivatives characterized by reproducible properties and derivatization degrees [26,27]. These polymers were then used for the production of both homopolymer (made of GelMA and CSMA alone) and heteropolymer (made of GelMA and CSMA combined at 1:1 weight ratio) cryogels, according to an already optimized procedure, in which cryogelation was initiated via APS and TEMED-mediated radical crosslinking, carried out at −12.0 ± 0.1 °C [6]. Both homopolymer and heteropolymer lyophilized cryogels showed a homogeneous, sponge-like morphology and a very fast swelling in PBS (pH = 7.4), as can be observed in Figure 1. According to the literature, most of the absorbed PBS is located in the macropores, which account for 66–71% of the total mass (Table S1) [36].

All the properties of the obtained cryogels were analyzed in comparison with Spongostan, a commercially available gelatin-based hemostyptic material, which has been used as potential three-dimensional scaffold for tissue regeneration [37].

**Figure 2:**
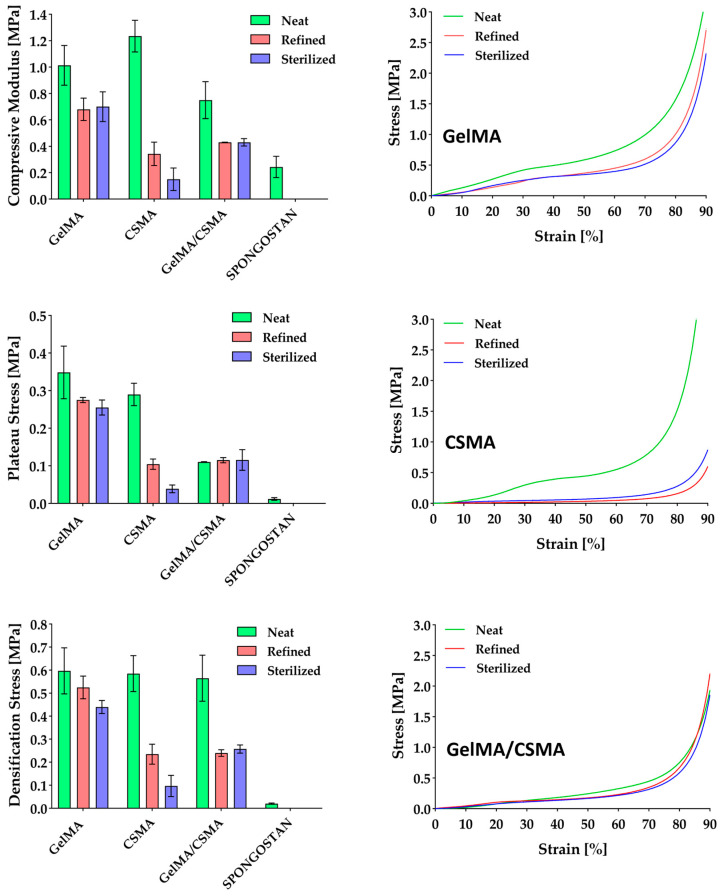
Compressive properties, i.e., modulus, plateau stress and densification stress, of Spongostan benchmark and GelMA, CSMA and GelMA/CSMA cryogels neat, refined and sterilized and the related compressive curves.

Cryogels’ compressive properties were evaluated and the results obtained from dry-condition tests are reported in Figure 2. All samples are characterized by compressive performance, i.e., modulus, plateau stress and densification stress, significantly higher than that of the commercial Spongostan. In particular, GelMA displays a stiffness three times higher, CSMA a stiffness four times higher and GelMA/CSMA a stiffness two times higher than Spongostan. Moreover, in dry conditions, there is a difference of more than one order of magnitude in plateau stress between Spongostan and all other samples proposed in the present work. These results are confirmed also in wet conditions, where GelMA displays a compressive modulus of 35 ± 7 kPa, CSMA of 45 ± 7 kPa and GelMA/CSMA of 15 ± 7 kPa, while Spongostan achieves only a 1.0 ± 0.3 kPa compressive modulus. 

**Figure 3:**
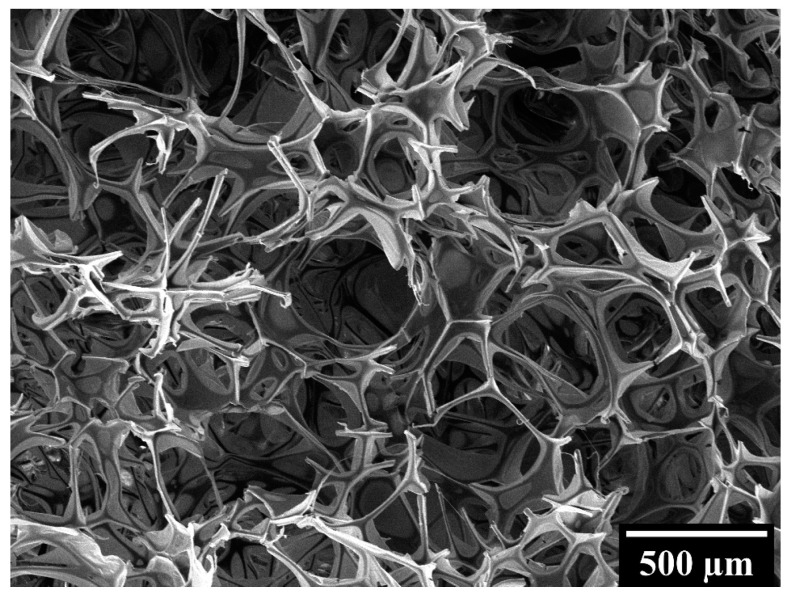
SEM micrograph of Spongostan benchmark.

**Figure 4:**
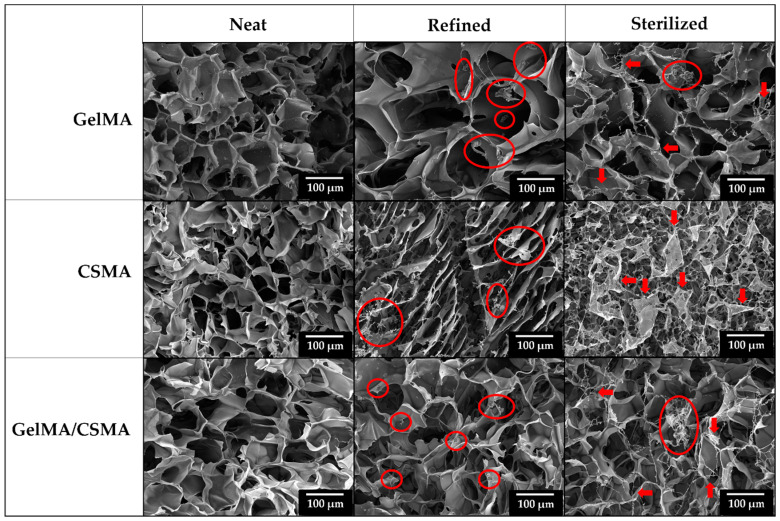
SEM micrographs of GelMA, CSMA and GelMA/CSMA cryogels neat, refined and sterilized. Red circles and red arrows evidence areas of microstructural changes within cryogel samples.

These differences in compressive behavior must be ascribed to the different microstructure of the biomaterials under consideration. In particular, Spongostan is characterized by an open-cell structure, as proved by the micrograph shown in Figure 3, while GelMA, CSMA and GelMA/CSMA display a close-cell structure, as proved by the micrographs in Figure 4. Closed cells are sealed off from their neighbors by membrane-like faces and entrap inside them air and gases, while open cells are interconnected and allow gases to flow, with a direct effect on material compressive response. In particular, open-cell foams deform primarily by cell-wall bending, and the fluids inside them are able to flow across their microstructure when the material is compressed. On the contrary, in closed-cell foams, cell-edge bending is accompanied by cell-face membranes stretching and, if they do not rupture, by the compression of the fluid which is trapped within the cells [38]. Both these phenomena are responsible for an increase in foam compressive properties.

Moving to the samples investigated in the present work, neat CSMA cryogels are characterized by the highest stiffness, while GelMA/CSMA displays the lowest one. The improved stiffness of CSMA over GelMA was already reported by Han et al. [33], who highlighted an increase from 13 kPa to 37 kPa in compressive modulus by adding chondroitin sulfate to gelatin-based cryogels. A similar effect was also observed in hydrogel scaffolds made of GelMA and CSMA [39]. This can be ascribed to the chemical structure of CS, which lends stiffness to the overall macromolecules [40].

The situation is different for GelMA/CSMA cryogels, in which a decrease of compressive modulus compared to GelMA can be observed, as already reported by Lai et al. for porous gelatin scaffolds with increasing content of CS [41]. This outcome can be likely ascribed to ionic interaction between the two polymers [42], which leads to segregation phenomena and provides an heterogeneous and scattered structure. Indeed, when GelMA and CSMA solutions are mixed to give a 1:1 weight ratio between the two polymers, an increase in the turbidity is observed measuring the transmittance at 600 nm, which confirms the occurrence of interaction between GelMA and CSMA, which may be responsible for the formation of an inhomogeneous network and also for the incomplete polymers crosslinking. As a matter of fact, while gel fractions higher than 0.90 were measured for GelMA and CSMA cryogels, this parameter decreases to 0.82 for GelMA/CSMA hybrid cryogels. This result indicates a poorly efficient chemical crosslinking between the two polymers under the adopted experimental conditions. This feature may also account for the faster degradation profile of the heteropolymer cryogels, compared to the homopolymer ones (Figure S1). The poor blending between GelMA and CSMA is also evidenced by the difference in the pore size distribution of heteropolymer and homopolymer cryogels. Indeed, heteropolymer cryogels showed a more scattered pore size distribution, with pore sizes mainly in the range of 5–110 μm and a mean pore size of 62.5 ± 20.4 μm (Table S1), whereas GelMA and CSMA cryogels displayed narrower pore size distributions (Figure S2), with mean pore sizes of 36.2 ± 6.2 μm and 51.6 ± 13.1 μm, respectively. 

Significant variations in the compressive behavior of the different cryogel scaffolds were observed after swelling and freeze-drying (refined samples) and also after sterilization. In particular, GelMA cryogels experience a decrease of almost 30.0% in stiffness, a decrease of 21.1% and 26.8% in the plateau stress and a decrease of 12.0% and 26.3% in the densification stress after refining and sterilization, while CSMA experience a decrease of almost 72.2% and 87.8% in stiffness, a decrease of 70.8% and 86.6% in the plateau stress and a decrease of 59.8% and 83.4% in the densification stress after refining and sterilization, respectively. The decrease in mechanical performance experienced by GelMA cryogels is much lower than that of the CSMA ones and can be mainly ascribed to the physical crosslinked part [43], which is unavoidably present despite the chemical free radical crosslinking, as reported by Di Muzio et al. [6]. Indeed, it has been reported that the combination of physical and chemical crosslinking allows the formation of GelMA networks characterized by high structural strength. In particular, physical interactions lead to ordered conformational structures, which cause a steep increase of the network stiffness, possibly resulting from higher total numbers of physical and chemical crosslinks [44,45]. 

More critical is the situation for CSMA cryogels, which experience a much stronger decrease in compressive properties after refining and sterilization. These outcomes can be likely ascribed to the high hydrophilicity of CSMA [46], which increases water affinity of the corresponding cryogels, as indicated by the higher swelling degree (Figure 1). This behavior leads to significant microstructural alterations following the water exposure in both refining and sterilization steps, as confirmed by SEM observation and modification of pore size distribution (Table S1). Indeed, SEM micrographs reported in Figure 4 show the appearance of conspicuous fibrillar structures (red circles) in the refined samples, while porous thin films across adjacent pores can be observed after sterilization (red arrows). 

Finally, intermediate results were observed with GelMA/CSMA heteropolymer cryogels, with a decrease of 42.6% in compressive modulus and a decrease of almost 55% in densification stress already after refining, whereas after sterilization they keep their properties almost unchanged. Considering that GelMA/CSMA cryogels experience a decrease in stiffness more pronounced than GelMA ones, it can be considered that CSMA hydrophilicity plays undoubtedly a significant role in compressive properties’ decrease. However, it can be noticed that the microstructural modifications of GelMA/CSMA cryogels observed in Figure 4 are more similar, even if more pronounced, to the ones reported for GelMA, which probably remains the main backbone of the cryogel. It is worth noting that even after sterilization, GelMA and GelMA/CSMA cryogels are characterized by mechanical properties better than those of neat Spongostan, with a compressive modulus 187.7% and 76.7% higher, respectively. 

**Figure 5:**
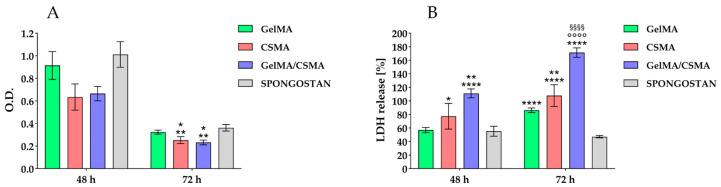
(**A**) MTS assay in tenocytes cultured on GelMA, CSMA, GelMA/CSMA and Spongostan for 48 and 72 h. The histogram represents the O.D. spectrophotometrically detected. ** vs. Spongostan *p* < 0.005, * vs. Gel-MA *p* < 0.05. (**B**) LDH assay of tenocytes cultured on GelMA, CSMA, GelMA/CSMA and Spongostan for 48 and 72 h. LDH released is reported as % LDH leakage. 48 h: **** vs. GelMA, Spongostan, *p* < 0.0001, ** vs. CSMA *p* < 0.005, * vs. Spongostan *p* < 0.05; 72 h: **** vs. Spongostan *p* < 0.0001, °°°° vs. GelMA *p* < 0.0001, ^§§§§^ vs. CSMA *p* < 0.0001, ** vs. GelMA *p* < 0.005. For both data values, the most representative of five different experiments is shown.

Then, the evaluation of the aforementioned cryogels was performed, from a biological point of view, by testing their biocompatibility in an in vitro model represented by tenocytes. Tenocytes were cultured on the above-described cryogel scaffolds up to 72 h. Metabolic activity, by means of MTS test, was read after 48 and 72 h of culture. After 48 h of culture, there are not statistically significant differences in the recorded metabolic activity values, while after 72 h of culture, the metabolic activity measured for tenocytes grown onto CSMA and GelMA/CSMA appear significantly affected with respect to that recorded on both GelMA and Spongostan (Figure 5A).

Then, the cytotoxicity exerted by the studied scaffolds on tenocytes was taken into consideration by measuring the LDH release, explanatory of cytotoxic stimuli, within the culture medium. After 48 h of culture, a statistically significant increase of cytotoxic response is detected for GelMA/CSMA with respect to all other tested biomaterials; in addition, an increase in LDH release is also recorded for CSMA with respect to Spongostan. After 72 h of culture, all tested scaffolds disclose a statistically appreciable increase of the cytotoxic response compared to Spongostan; a statistical significance of LDH values obtained from CSMA and GelMA/CSMA with respect to GelMA is additionally recorded (Figure 5B). The same trend, in terms of cell metabolic activity and cytotoxicity, is detected in a parallel experimental model obtained by seeding and culturing primary HGFs on the same tested scaffolds (Supplementary Materials, Figure S3).

**Figure 6:**
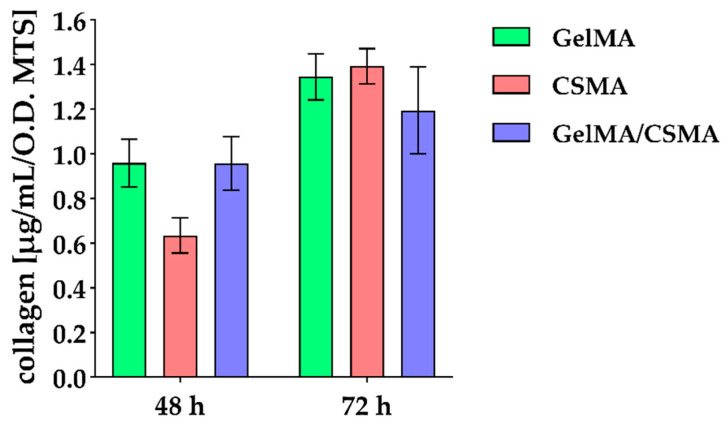
Collagen type I secretion in tenocytes cultured on GelMA, CSMA and GelMA/CSMA cryogels for 48 and 72 h. Secretion levels are reported as µg/mL per MTS O.D. values. The results are the mean ± SD of three samples; the most representative of three different experiments is shown.

Col I secretion has been also evaluated, with a reduction in protein release in the presence of CSMA found after 48 h of culture, whereas, after 72 h of culture, a slight decrease in the presence of GelMA/CSMA can be identified (Figure 6).

**Figure 7:**
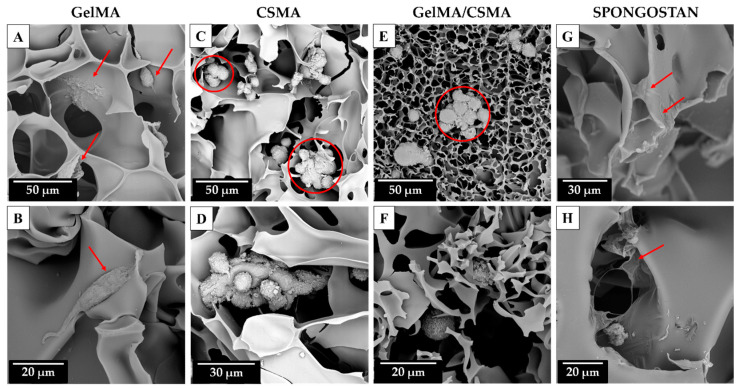
SEM morphological analysis of tenocytes cultured on GelMA ((**A**,**B**), magnification 1750× and 3800, respectively), CSMA ((**C**,**D**), magnification 1550× and 2850, respectively), GelMA/CSMA ((**E**,**F**), magnification 1650× and 4100, respectively) cryogels and Spongostan ((**G**,**H**), magnification 2200× and 3000, respectively) after 72 h of culture. Red arrows indicate adhered cells, while red circles indicate dead grouped cells.

To conclude, a morphological analysis of the above-mentioned cryogel scaffolds in the presence of tenocytes after 72 h of culture was carried out by means of SEM. The morphological analysis shows that a tentative adhesion of tenocytes on GelMA cryogel is detectable even if it can be also evidenced that, for some cells, being round-shaped on the biomaterial surface, the adhesion process is partially impeded due to the physical, chemical or physicochemical characteristics of the surface (Figure 7A,B, red arrows). 

A better morphological situation is recorded in the presence of Spongostan biomaterial, as tenocytes appear totally flattened on the available surface, thus leading to an undisturbed process of adhesion (Figure 7G,H, red arrows). Conversely, a deeply affected process of adhesion is evidenced on CSMA and on GelMA/CSMA cryogel scaffolds. Both reveal completely round-shaped grouped dead cells, highlighting impassable difficulties for tenocytes in the early and late phases of the attachment on the available surface (Figure 7C–F, red circles). It could be also hypothesized that, especially for the hybrid scaffold GelMA/CSMA, the inhomogeneous structure revealed by SEM analysis could be also responsible for the poor cell performance.

The poor performance of CSMA-based cryogels is in line with the results reported in the literature [47,48] and could be dependent on the scaffold composition, but also on the mechanical properties. A general worsening of these properties was observed following the sterilization of the cryogel samples, which could have provided inadequate mechanical signaling to regulate cell fate. However, the use of CSMA alone could be an inappropriate strategy to allow proper cell adhesion to the scaffold. Indeed, according to the literature, CSMA-based cryogels are frequently modified, introducing cell attachment sequences, such as RGD motif, or combined with other biopolymers in order to improve and promote the cell adhesive properties of the scaffold [49,50]. Nevertheless, the hybrid network based on the combination of CSMA with GelMA also showed poor biological behavior, despite the RGD motifs inherently present on Gel backbone. Therefore, although it is reported that ECM-mimicking cryogels can successfully support the cell adhesion process and the accumulation of cartilage-specific ECM productions, thus having great potential in the tissue engineering field, particularly cartilage tissue engineering, more investigations are needed to find more efficient ways to develop hybrid scaffolds using these components. In this sense, double-network structures could be a strategy to help combine different biopolymers, while creating mechanically resistant hybrid scaffolds [51]. Moreover, the results obtained in this work highlight the importance of evaluating the ability of cryogel scaffolds to withstand an efficient sterilization, while maintaining the scaffold performance in an acceptable range of properties. This evaluation is of fundamental importance when cryogels are proposed as scaffolds for tissue engineering applications [22,52]. The impact of the sterilization process on the stability of cryogels should be carefully considered and investigated when considering clinical translation.

## 4. Conclusions

Methacryloyl derivatives of Gel and CS were used to develop ECM-mimicking scaffolds through the cryogelation technique. Both homopolymer and heteropolymer scaffolds were developed via radical-mediated crosslinking reaction; however, the hybrid polymer network did not allow us to take advantage of the properties of the building polymers, Gel and CS. Indeed, despite the presence of Gel, the hybrid GelMA/CSMA scaffold did not allow proper cell adhesion and showed a general worsening of the biological behavior, compared to homopolymer GelMA-based cryogels. This outcome could be ascribed to inadequate topography or mechanical properties of the cryogel scaffold, especially following the sterilization process, probably deriving from poor polymer mixing and consequent segregation phenomena within the hybrid network. Although cryogels represent a valid platform as scaffolds for tissue engineering, further investigations are needed for the development of efficient hybrid cryogels, in order to properly take advantage of the features of all the building components. In particular, the effect of the sterilization process on the scaffold properties should be adequately assessed. This suggests that the structural stability of hybrid cryogel for tissue engineering applications is of particular importance when considering clinical translation. 

## Data Availability

The original contributions presented in the study are included in the article/Supplementary Material, further inquiries can be directed to the corresponding author.

## Supplementary Materials

The following supporting information can be downloaded at: https://www.mdpi.com/article/10.3390/polym16131939/s1. Figure S1. Degradation profiles of homopolymer and heteropolymer cryogels determined in PBS (pH = 7.4) at 37.0 ± 0.5 °C. Figure S2. Pore size distribution of cryogel scaffolds, evaluated through the SEM micrographs analyzed with the image processing software Image J. Table S1. Pore size distributions, mean pore diameters and porosities of cryogel scaffolds. Figure S3. (A) MTS assay in primary HGFs cultured on GelMA, CSMA, GelMA/CSMA and Spongostan for 48 and 72 h. The histogram represents the O.D. spectrophotometrically detected. (B) LDH assay of primary HGFs cultured on GelMA, CSMA, GelMA/CSMA and Spongostan for 48 and 72 h. LDH released is reported as % LDH leakage. 48 h: the most representative of five different experiments is shown for both data values.

## Abbreviations

Gel, gelatin; GelMA, methacryloyl gelatin; CS, chondroitin sulfate; CSMA, chondroitin sulfate methacrylate; HGFs, human gingival fibroblasts; SEM, scanning electron microscopy; LDH, lactate dehydrogenase; O.D., optical density; Col I., Collagen I.
