# Supplementary material for: Impact of Composition and Autoclave Sterilization on the Mechanical and Biological Properties of ECM-Mimicking Cryogels
Source: Polymers (Basel). 2024 Jul 7;16(13):1939. doi: 10.3390/polym16131939 (PMC11244042; doi:10.3390/polym16131939)
Supplement: Supplementary file 1 [file polymers-16-01939-s001.zip › polymers-3046651-supplementary.pdf]

Impact of composition and autoclave sterilization on the mechanical and biological properties of ECM-mimicking cryogels

*Laura Di Muzio[1], Susi Zara[2], Amelia Cataldi[2], Claudia Sergi[3], Vito Cosimo Carriero[1], Barbara Bigi[1], Simone Carradori[2], Jacopo Tirillò[3], Stefania Petralito[1], Maria Antonietta Casadei[1], Patrizia Paolicelli[1]**

[1] Department of Drug Chemistry and Technologies, Sapienza University of Rome, 00185 Rome, Italy

[2] Department of Pharmacy, University "G. d'Annunzio" of Chieti-Pescara, 66100 Chieti, Italy

[3] Department of Chemical Engineering Materials Environment, Sapienza University of Rome, 00184 Rome, Italy

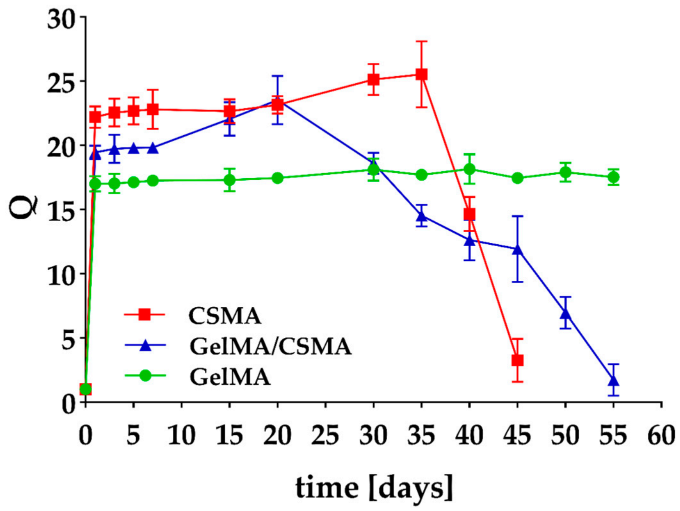

Figure S1. Degradation profiles of homopolyer and heteropolymer cryogels in PBS (pH = 7.4) at 37.0 ± 0.5 °C.

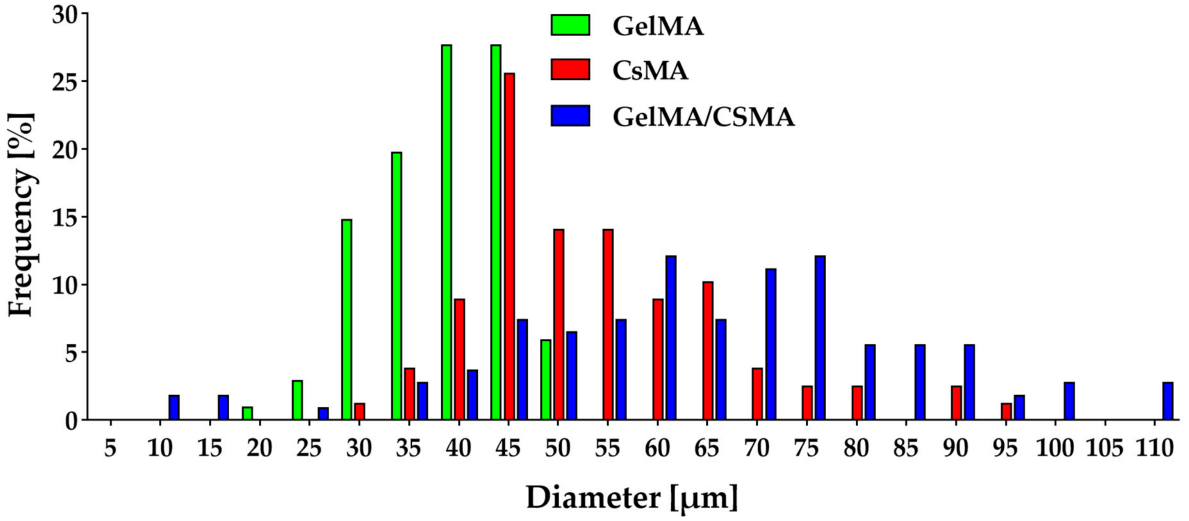

Figure S2. Pore size distribution of cryogel scaffolds, evaluated through the SEM micrographs analyzed with the image processing software Image J. A total number of 200 pores was measured to obtain a statistically significant distribution.

Table S1. Pore size distributions, mean pore diameters and porosities of cryogel scaffolds.

Cryogel	Treatment	Pore size distribution (µm)	Mean pore diameter (µm)	Porosity (%)
GelMA	None	15-50	36.2 ± 6.2	66 ± 1
	Refinement	35-110	70.6 ± 20.3	
	Sterilization	45-105	71.5 ± 11.6	
CSMA	None	25-95	51.6 ± 13.1	71 ± 1
	Refinement	30-105	58.3 ± 15.4	
	Sterilization	30-105	63.3 ±19.2	
GelMA/CSMA	None	5-110	62.5 ± 20.4	68 ± 1
	Refinement	45-100	68.9 ± 14.5	
	Sterilization	40-110	75.4 ± 19.1	

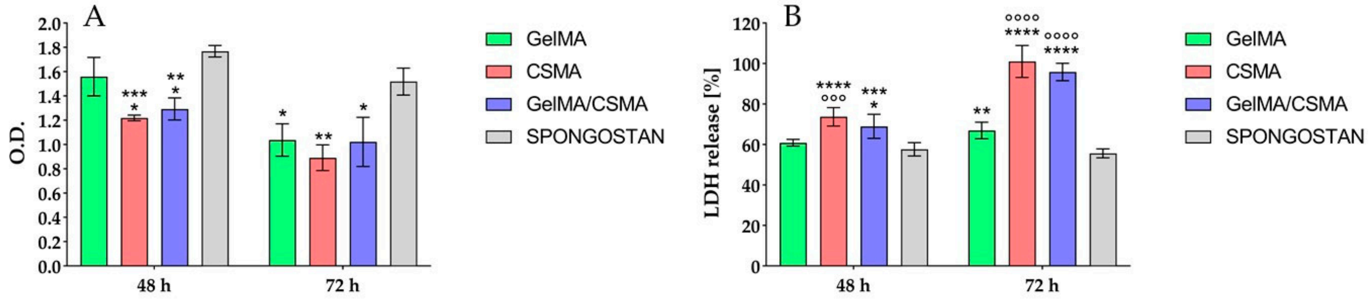

Figure S3. A) MTS assay in primary HGFs cultured on GelMA, CSMA, GelMA/CSMA and Spongostan for 48 and 72 h. The histogram represents the O.D. spectrophotometrically detected. B) LDH assay of primary HGFs cultured on GelMA, CSMA, GelMA/CSMA and Spongostan for 48 and 72 h. LDH released is reported as % LDH leakage. 48 h: the most representative of five different experiment is shown for both data values.
